# Advances in electromyography armbands for gesture recognition and multimodal fusion

**DOI:** 10.1016/j.isci.2025.114517

**Published:** 2025-12-24

**Authors:** Ruihao Zhang, Yingping Hong, Helei Dong, Xiong Yang, Huixin Zhang, Lizhi Dang

**Affiliations:** 1 School of Semiconductor and Physics, North University of China, Taiyuan, China; 2 School of Instrument and Electronics, North University of China, Taiyuan, China; 3 Department of Electronic and Computer Engineering, The Hong Kong University of Science and Technology, Hong Kong, China

**Keywords:** electrical engineering, bioengineering, health science

## Abstract

Surface electromyography (sEMG) signals carry abundant information regarding human motion and muscle activity, and armbands equipped with sEMG acquisition can decode gestures via pattern recognition algorithms. Consequently, sEMG armbands have been increasingly adopted for building natural and efficient human-machine interfaces. With the expansion of datasets, rapid hardware iteration, and the emergence of multiple sensing modalities, it has become essential to systematically examine how different armband designs and integration strategies affect recognition performance. This paper systematically reviews the architectures and technical specifications of mainstream sEMG armbands and compares the integration and performance of additional modalities—such as inertial measurement unit (IMU), force myography (FMG), magnetomyography (MMG), sonomyography (SMG), near-infrared spectroscopy (NIRS), light myography (LMG), and electrical impedance tomography (EIT)—within sEMG-based systems. This review also highlights the conceptual value of multimodal fusion for improving robustness and generalizability and outlines directions for developing lightweight, low-power, and cost-effective armbands that better support complex human-machine interaction scenarios.

## Graphical abstract

**Figure:**
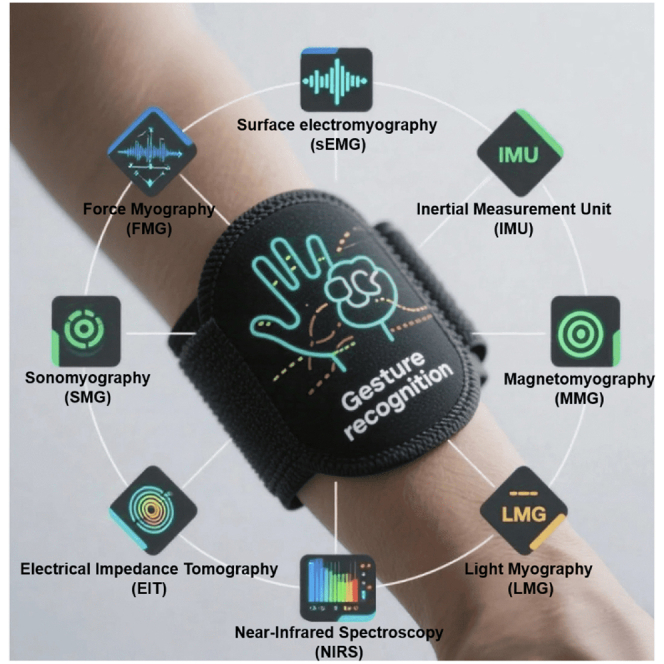

## Introduction

With the rapid development of intelligent interaction systems and wearable technologies, how to efficiently and reliably perceive human motion intention has become a core technological challenge in fields such as human-computer interaction (HCI), assistive rehabilitation, and virtual reality.^1^^,^^2^^,^^3^

Surface electromyography (sEMG) signals can directly reflect muscle activation status,^4^ contraction intensity,^5^ and movement intention,^6^ making them one of the most promising signal sources for acquiring motor intent.^7^^,^^8^^,^^9^ In recent years, wearable devices based on sEMG, particularly sEMG armbands, have gained widespread attention for application in gesture recognition,^10^^,^^11^^,^^12^ motion monitoring,^13^^,^^14^^,^^15^ and intelligent control^16^^,^^17^^,^^18^ due to their non-invasive nature, rapid response, and flexible design. Compared with traditional electrode systems, sEMG armbands provide a compact, integrated solution that combines multi-channel signal acquisition, power management, wireless communication, and signal processing into a single device. This integration strikes a balance between wearability and system performance, significantly enhancing their practical utility.

With advancements in hardware design and material technologies, the system architecture of sEMG armbands has been continuously optimized, and electrode materials have been progressively improved. Combined with front-end circuit designs featuring adjustable bandwidth, sampling rate, and gain, these developments have significantly enhanced the sensitivity and stability of signal acquisition. At the same time, ongoing progress in signal processing and pattern recognition algorithms has led to continuous improvements in gesture recognition accuracy and response speed.^19^^,^^20^^,^^21^ In parallel with functional enhancements, optimizing for low power consumption, miniaturization, cost-effectiveness, and ergonomic design has become a key direction in the evolution of sEMG armband systems. Meanwhile, the increasing availability of large-scale sEMG datasets has greatly promoted the development and validation of more robust recognition algorithms.

However, single-modal sEMG still faces certain limitations in practical applications.^22^^,^^23^^,^^24^ For example, in scenarios such as complex gesture recognition, static gesture detection, cross-user generalization, and deep muscle sensing, sEMG signals are susceptible to factors like muscle fatigue, variations in skin resistance, and electrode contact quality, which can reduce system robustness.^25^^,^^26^^,^^27^ As a result, relying solely on sEMG signals is often insufficient to meet the increasing demands of complex human-machine interaction tasks. To address these challenges, many studies have explored integrating sEMG with other physiological or motion-sensing modalities.^28^^,^^29^^,^^30^ These efforts have shown some progress and suggest a promising direction for improving system performance.

Considering the rapid development of wearable sEMG armbands for hand gesture recognition, it is important to systematically review existing research from both device- and system-level perspectives. Although numerous review papers have been published in the field of sEMG, most focus on pattern recognition algorithms^31^^,^^32^^,^^33^ or signal processing techniques,^34^^,^^35^^,^^36^^,^^37^ rather than the practical implementation of wearable systems. Several studies have explored multimodal fusion approaches.^38^^,^^39^^,^^40^ However, these studies mainly discuss fusion methodologies in general contexts without emphasizing their integration into sEMG-based armband platforms (where integration must consider power, cost, stability, and wearability). Some reviews have examined wearable sEMG devices.^13^^,^^18^^,^^41^^,^^42^^,^^43^ Yet, they do not specifically focus on armband-type systems for gesture recognition, which leads to the omission of many recent advances. In contrast, this paper presents a comprehensive, application-oriented review of sEMG armband systems for hand gesture recognition, focusing on hardware technologies, multimodal fusion, and limitations and potential solutions. The aim is to bridge the gap between fundamental research and system-level implementation, providing a unified reference for both academic exploration and the engineering design of next-generation sEMG armband systems.

### Search strategy

The paper search and selection process followed the AMSTAR guidelines to ensure methodological transparency and reproducibility. The search aimed to identify published studies related to sEMG armbands and multimodal fusion for gesture recognition from 2010 to 2025, covering both foundational developments and recent advances. Electronic databases, including IEEE Xplore, Science Direct, Springer Link, MDPI, and Frontiers, as well as several additional sources, were systematically queried. The search was restricted to English-language publications and included journal articles, conference papers, and review papers.

The initial search employed the keyword “sEMG” to capture a comprehensive range of electromyography-related research. Subsequently, within these retrieved records, a secondary screening was conducted using the additional keywords “armband,” “bracelet,” and “modal fusion” to further narrow the scope toward studies on wearable gesture recognition systems. The retrieved records were screened by title and abstract to exclude unrelated studies.

**Figure 1:**
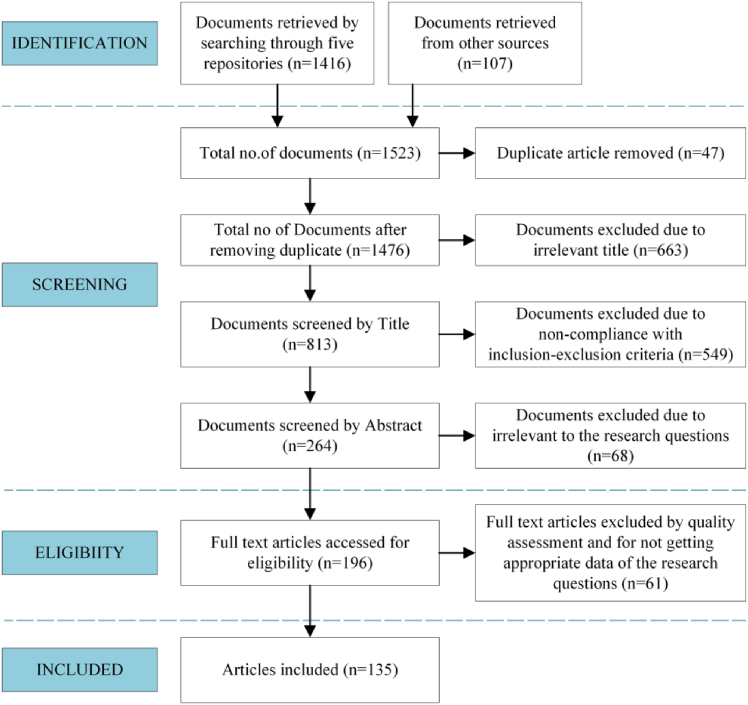
PRISMA flow diagram of searching the primary document

The inclusion criteria required that the work involved the design, implementation, or evaluation of sEMG-based armbands, or provided technical or methodological insights applicable to armband-based gesture recognition, such as multimodal sensor fusion or wearable system design. Studies unrelated to wearable sensing or lacking direct relevance to gesture recognition were excluded. After manual review and cross-checking to remove duplicates, 135 representative publications were identified as the main research basis of this review; they were distributed across IEEE Xplore (42), Science Direct (31), Springer Link (22), MDPI Sensors (17), Frontiers (5), and other sources (19). The PRISMA flow diagram in Figure 1 provides a visual summary of the entire screening process.

## Overview and technical features of sEMG armband products

### Composition of the sEMG armband

An sEMG armband primarily consists of an electrode system, front-end analog circuitry, an analog-to-digital converter (ADC), a microprocessor, a wireless communication module, a power management system, a wearable structure, and optional extension modules. The electrode system attaches to the skin surface to collect sEMG signals; the front-end analog circuitry amplifies and filters these signals; the ADC converts the analog signals into digital form; the microprocessor handles data acquisition, processing, and control; the wireless communication module transmits data to external devices; the power management system supplies power and manages the battery; the wearable structure ensures a secure and comfortable fit; and optional modules—such as inertial measurement units (IMUs), storage units, additional modality sensors, and feedback devices—enhance system functionality and user interaction.

### Progress in sEMG armband technology

**Figure 2:**
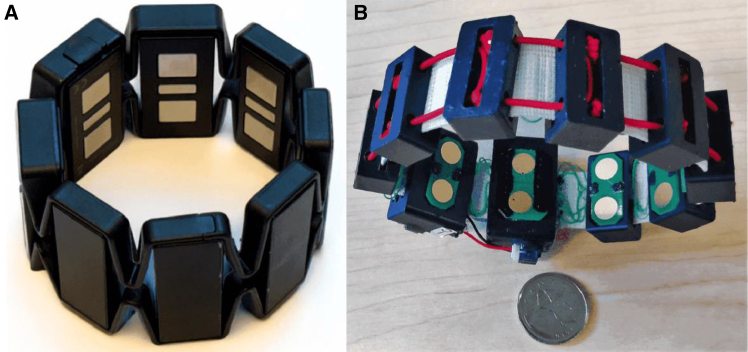
Myo armband and 3DC armband (A) Myo armband, reproduced from Grattarola et al.^49^ under the Creative Commons Attribution (CC BY 4.0) license. (B) 3DC armband, reproduced from Côté-Allard et al.^50^ under the Creative Commons Attribution (CC BY 4.0) license.

One of the earliest and most mature sEMG armband products can be traced back to 2013, when Thalmic Labs launched the Myo armband, as shown in Figure 2A. The Myo is equipped with a low-power ARM Cortex-M4 microprocessor (MK22FN1M0, 120 MHz), an eight-channel sEMG electrode array, and an IMU (MPU9150), and it incorporates the TSZ124 quad operational amplifier from STMicroelectronics.^44^ The device features a sampling rate of 200 samples per second (sps), a bandwidth of 5–100 Hz, and an 8-bit ADC. Its retail price was approximately $200. The Myo armband has been widely demonstrated to be effective for gesture recognition.^45^^,^^46^ Sayin et al. collected sEMG signals using the Myo armband,^47^ extracted features such as mean absolute value and slope sign changes, and employed an artificial neural network (ANN) to classify five types of hand movements. The experiments were conducted on five intact users, achieving an average classification accuracy of 88.4%. Cengiz Tepe acquired sEMG data using the Myo armband and applied an ANN method to recognize six types of static gestures, achieving an average accuracy of 94.4%.^48^

Côté-Allard et al. designed an sEMG armband named 3DC,^50^ as shown in Figure 2B. The 3DC armband uses a 0.13-μm CMOS system-on-chip (SoC) developed by Gagnon-Turcotte et al.^51^ The MCU (Microcontroller Unit) is the MSP430F5328 from Texas Instruments (TI), USA. The 3DC armband features 10 sEMG acquisition channels, a 10-bit ADC resolution, a sampling rate of 1,000 sps, and a bandwidth of 20–500 Hz, with an estimated cost of about $200. Côté-Allard et al. conducted a comparative experiment in which both the Myo and 3DC armbands were worn simultaneously on the dominant arms of 22 intact users. The participants performed 11 static gestures movements, each lasting 10 s or longer, while sEMG signals were recorded. Using convolutional neural network (CNN) for sEMG signal recognition and classification, the 3DC and Myo armbands achieved average accuracies of 89.7% and 86.41%, respectively, in the experiment. It can be seen that increases in the number of channels, sampling rate, and resolution of sEMG armbands have a positive impact on gesture recognition accuracy. However, the 3DC armband transmits the data collected by its 10-bit ADC in a 16-bit format, resulting in some waste of system resources.

**Figure 3:**
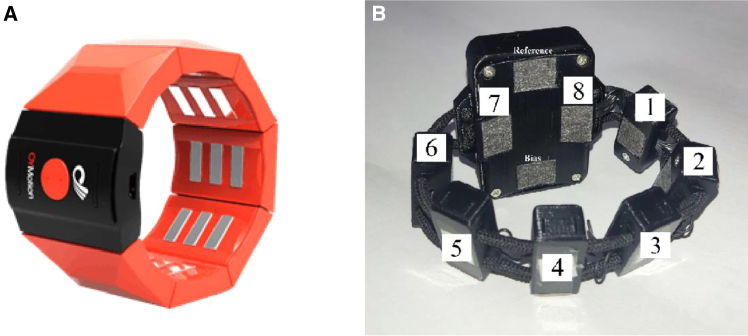
gForce-Pro+ armband and MindRove armband (A) gForce-Pro+ armband, reproduced from Zhu et al.^54^ under the Creative Commons Attribution (CC BY 4.0) license. (B) MindRove armband, reproduced from Concha-Pérez et al.^55^ under the Creative Commons Attribution (CC BY 4.0) license.

Figure 3A shows the gForce-Pro+, an sEMG armband developed by OYMotion. It features eight acquisition channels, with a maximum sampling rate of 500 Hz at 12-bit ADC resolution and 1 kHz at 8-bit ADC resolution. Wireless communication is implemented via Bluetooth BLE 4.0. The gForce-Pro incorporates an edge AI computing module, enabling users to contribute their own sEMG signals to a centralized database. These signals are used to train intention recognition models via OYMotion’s proprietary algorithms, which can be applied to control the company’s OHand intelligent bionic hand. The current version of the smart algorithm can recognize eight distinct motor intentions, while the mechanical system supports up to 18 expandable gestures. The gForce-Pro+ is priced relatively high, at approximately $1,250. The gForce-Pro+ demonstrates excellent performance in gesture recognition tasks. Campbell et al.^52^ conducted a five-gesture dynamic classification experiment using the gForce-Pro+, achieving an average recognition accuracy of up to 92.4%. Uimonen et al.^53^ performed a six-gesture static classification experiment with the gForce-Pro+, reporting an average F1 score of 0.94.

Figure 3B shows the sEMG armband developed by the Hungarian company, MindRove. This armband includes eight sEMG electrodes, features a 24-bit ADC resolution and a sampling rate of 500 Hz, and transmits data to a computer via Wi-Fi. It can operate continuously for 4 to 6 h and is priced slightly higher, at $729. The MindRove sEMG armband has also been proven effective for sEMG detection. Taori and Lim^56^ used the MindRove sEMG armband to collect sEMG signals, detect the start and end of each lifting and lowering activity through continuous sEMG measurement, and classify hand loads. The average detection accuracy ranged from 79.2% to 86.9%, indicating substantial scope for improvement. In the study performed by Köllőd et al.,^57^ electromyography (EMG) signals were collected from six intact users performing seven static gestures. Extra Trees, Random Forest, and EEGNet achieved average accuracies of 59.68%, 58.88%, and 56.77%, respectively, showing relatively low performance. An earlier study^55^ also indicates that the MindRove armband shows mediocre performance in hand gesture recognition at the current stage.

**Figure 4:**
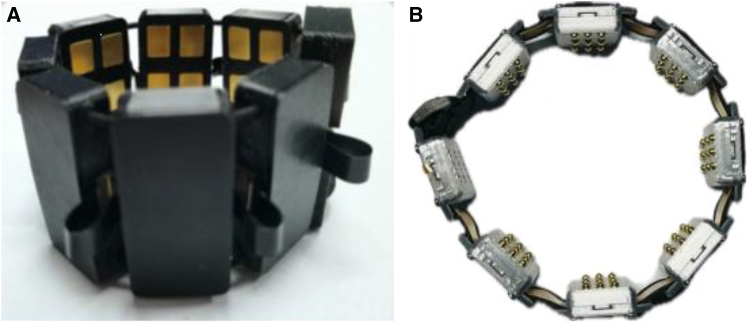
α armband and Medium-density EMG armband (A) α armband, reproduced from Zhang et al.^58^ under the Creative Commons Attribution (CC BY 4.0) license. (B) Medium-density EMG armband, reproduced from Aghchehli et al.^60^ under the Creative Commons Attribution (CC BY 4.0) license.

Figure 4A shows an sEMG armband proposed by Zhang et al.,^58^ referred to as the α armband. The analog front-end of the α armband employs the RHD2216 chip (Intan Technologies, USA), while the MCU is a high-performance ARM Cortex-M7 controller (STM32F765VIH6). The device features 16 sEMG acquisition channels, 16-bit ADC resolution, an adjustable bandwidth ranging from 0.1 to 20 kHz, and a maximum adjustable sampling rate of 2,000 sps per channel. The high sampling rate enables the waveform to more closely resemble the original signal, which benefits accurate sEMG recognition and classification.^59^ Moreover, the adjustable bandwidth and sampling rate allow the α armband to flexibly handle various sEMG acquisition tasks by filtering out irrelevant frequency components and adapting to different operating conditions. From both time-frequency and channel-number perspectives, six sets of comparative experiments were conducted using CNNs for training and testing. The average recognition accuracy, evaluated on 30 intact users performing 10 static gestures, ranged from 88.6% to 98.6%.

Aghchehli et al.^60^ introduced a medium-density EMG system that addresses limitations in sEMG control by integrating 21 digital electrodes into a compact armband (electrodes: Mill-Max Manufacturing Corporation, Surface Mount Spring-Loaded Pin, part no. 0945-0-15-20-09-14-11-0, 2024). This design improves decoding performance while maintaining the compact form factor typical of low-density systems. As shown in Figure 4B, the armband consists of one main node and seven sensor nodes. Each sensor module includes an analog front-end and a signal-processing subsystem. The analog front-end (ADS1293, TI) features a fixed-gain preamplifier, a 24-bit ADC across three channels, and an integrated EMI filter. Each channel connects to two active electrodes and one reference electrode. The processing unit is powered by a 32-bit ARM Cortex-M4 microcontroller (STM32L433RCI3, STMicroelectronics) connected to the analog front-end via a 16 MHz SPI. It samples sEMG signals at 1,067 Hz. A second-order IIR Butterworth band-pass filter (30–350 Hz) removes unwanted spectral components, and a notch filter (50/60 Hz) eliminates power-line interference. Recognition tests on 11 intact users performing 6 static gestures achieved 97.8% accuracy using temporal convolutional networks (TCNs).

**Figure 5:**
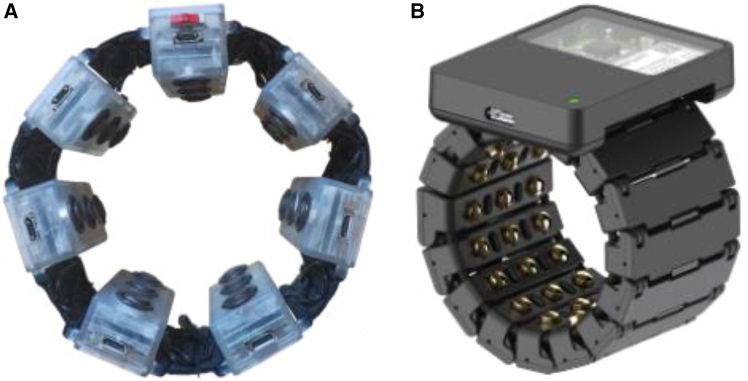
7-channel sEMG armband and sEMG-RD armband (A) 7-channel sEMG armband, reproduced from Mongardi et al.^63^ under the Creative Commons Attribution (CC BY 4.0) license. (B) sEMG-RD armband, reproduced from Kaifosh et al.^64^ under the Creative Commons Attribution (CC BY 4.0) license.

Figure 5A shows an sEMG armband proposed by Mongardi et al.^61^ The analog front-end of the armband includes input and output protection, a decoupling circuit (using the voltage follower TLV8542 to provide proper impedance for the amplifier input and ensure the integrity of the sEMG signal), a two-stage gain-adjustable amplification circuit (composed of INA333 and LPV821), a high-pass filter, and a low-pass filter (preserving the signal range of 30–400 Hz).^62^ The MCU is the Apollo3 Blue, based on the ARM Cortex-M4F core. In an online experiment^63^ with 25 intact users performing nine static gestures, the system achieved an average prediction accuracy of 93.36%.

Tam et al.^65^^,^^66^ presented a real-time fine-grained hand gesture recognition system, referred to as HD-sEMG. The HD-sEMG system adopts high-quality, low-power components, including the RHD2132 electrophysiological analog front-end and ADC from Intan Technologies. This integrated circuit (IC) allows simultaneous recording of 32 amplified electromyography channels using a 16-bit successive approximation ADC. The data are acquired via SPI using an ultra-low-power MSP430F5529 microcontroller from TI and then transmitted using an ultra-low-power 2.4 GHz transceiver nRF24L01+ from Nordic Semiconductor. HD-sEMG employs an embedded CNN to classify hand muscle contractions sensed from the forearm. In an offline experiment with a single intact user performing eight static gestures, the system achieved an average accuracy of 98.15%. In the online experiment with six static gestures, the average accuracy reached 96.83%.

Figure 5B shows the sEMG armband developed by Ctrl-labs, a company under Meta, named sEMG-RD.^64^ The sEMG-RD uses 48 pogo-pin circular electrodes to provide good comfort and contact quality. The 48 channels are configured as 16 bipolar channels arranged along the proximal and distal regions, with the remaining electrodes used for shielding or grounding. Each electrode has a diameter of 6.5 mm (gold-plated brass). For each differential sensing channel, the center-to-center spacing between paired sensing electrodes is 20 mm. The sEMG-RD features low-noise analog sensors with an input-referred RMS noise of 2.46 μVrms. The analog sensor has a nominal gain of 190, an ADC resolution of 12 bits, and a full-scale range of 2.5 V, providing approximately 65.5 dB of dynamic range. Each channel is sampled at 2,000 Hz. Trained on neuromotor data from over 6,000 participants, the Ctrl-labs model generalized well without user-specific calibration, achieving over 90% accuracy for handwriting and gesture recognition and over 75% for wrist posture classification on users.

Recently, Meta introduced a new sEMG armband called Orion (source: https://www.meta.com/blog/orion-ar-glasses-augmented-reality/). According to Meta’s description, the armband utilizes sEMG signals and IMU data to recognize gestures such as finger pinches, fist clenching, thumb taps, and sliding movements, which are used to control and manipulate virtual objects. However, the specific hardware design and performance specifications have not been disclosed, making it difficult to objectively evaluate the system’s actual performance.

In addition to these, numerous other noteworthy sEMG armbands or wearable sEMG devices exist. However, due to limited technical details, suboptimal ergonomics, or high cost, this review provides only a brief overview. Kartsch et al.^67^ designed an sEMG wristband powered by a small flexible solar panel, achieving 94.02% accuracy in a five-gesture recognition experiment. Mongardi et al.^68^ developed a real-time, low-power embedded system. Participants performed five gestures across five sessions, achieving 96.34% accuracy, with a peak latency of 8.5 ms and an average power consumption of 0.8 mW. Bi et al.^69^ proposed a wearable system called the electromyography bridge (EMGB), including an eight-channel sEMG detection armband and a four-channel constant-current stimulation armband, for multi-gesture rehabilitation of paralyzed limbs, achieving over 90% classification accuracy. Xu et al.^70^ presented an sEMG armband with onboard training and real-time decoding algorithms, capable of controlling a six-degree-of-freedom prosthetic arm to perform tasks such as making phone calls.

Guo et al.^71^ introduced an sEMG armband with a group-training framework, allowing pre-training on multiple users and testing on new users. Without individual training, it achieved 85% classification accuracy across seven gestures. Ergeneci et al.^72^ proposed an embedded, eight-channel, wireless, noise-canceling system with adaptive muscle contraction detection, achieving up to 98.98% accuracy. Wu et al.^73^ developed a wearable system for real-time recognition of American Sign Language by fusing IMU and sEMG data. Using information-gain-based feature selection and an SVM (Support Vector Machine) classifier, average accuracies were 96.16% (intra-subject) and 85.24% (cross-session). Yang et al.^74^ introduced a wearable armband with a novel feature selection method, recognizing all nine targeted hand gestures with 96.20% average accuracy. Moin et al.^75^ reported a wearable sEMG system with screen-printed electrodes and in-sensor adaptive learning. Two participants performed 13 gestures with 97.12% accuracy; when extended to 21 gestures, accuracy remained 92.87%, and model updates improved performance by 9.5% without extra computation. Other studies have also proposed several self-designed sEMG armbands or wearable sEMG devices,^76^^,^^77^^,^^78^^,^^79^^,^^80^^,^^81^^,^^82^^,^^83^^,^^84^^,^^85^^,^^86^ but these systems offer no particular advantages over existing products.

In summary, sEMG armbands for natural, intention-driven HCI are gradually moving from laboratory research to practical applications, showing significant potential in intelligent control, virtual reality, and rehabilitation. As datasets expand on a large scale and AI algorithms continue to advance, sEMG armbands are expected to play an increasingly important role in enabling seamless human-machine integration.

### Comparison of sEMG armbands

Table 1 summarizes the published studies conducted using each representative armband, along with their experimental performance in gesture recognition. Table 2 presents a technical comparison of the nine sEMG armbands mentioned earlier: ADC refers to analog-to-digital converter; sps refers to samples per second; IMU denotes inertial measurement unit; 9-axis indicates an accelerometer, gyroscope, and magnetometer, while 6-axis refers to an accelerometer and gyroscope; BLE stands for Bluetooth Low Energy; and power consumption refers to battery life or device power consumption.

**Table 1:** Performance comparison of sEMG armbands

sEMG Armband	Reference	Gesture recognition capability
Myo	Hu et al.^44^; Lv et al.^45^; Pizzolato et al.^46^; Grattarola et al.^49^; Sayin et al.^47^; Tepe and Erdim^48^	offline test: 10 intact users, 6 static gestures, and 94.4% average accuracy online test: 10 intact users, 6 static gestures, 94% average accuracy, and 338 ms latency
3DC	Côté-Allard et al.^50^; Gagnon-Turcotte et al.^51^	offline test: 22 intact users, 11 static gestures, and 89.47% average accuracy
gForce-Pro+	Campbell et al.^52^; Uimonen et al.^53^; Zhu et al.^54^	offline test: 16 intact users, 5 dynamic gestures, and 92.4% average accuracy
MindRove	Taori and Lim^56^; Köllőd et al.^57^; Concha-Pérez et al.^55^	offline test: 9 intact users, 3 dynamic gestures, and 79.2%–86.9% average accuracy
α	Zhang et al.^58^	offline test: 30 intact users, 10 static gestures, and 88.6%–98.6% average accuracy
Medium-density EMG armband	Aghchehli et al.^60^	offline test: 11 intact users, 6 static gestures, and 97.8% average accuracy
7-channel sEMG armband	Mongardi et al.^61^; Rossi et al.^62^; Mongardi et al.^63^	online test: 25 intact users, 9 static gestures, 93.36% average accuracy, 181.75 ms latency, and 2.92 mA average current consumption
HD-sEMG	Tam et al.^65^; Tam et al.^66^	offline test: 1 intact user, 8 static gestures, and 98.15% average accuracy online test: 1 intact user, 6 static gestures, 96.83% average accuracy, 200 ms latency, and 49.5 mW average power consumption
sEMG-RD	Kaifosh et al.^64^	offline test: 4,800 intact users, 9 dynamic gestures, and 90% average accuracy

**Table 2:** Performance comparison of sEMG armbands

Armbands	Myo	3DC Paper^50^	gForce-Pro+ Paper^52^	MindRove Paper^56^	α Paper^58^	Paper^60^	Paper^61^	HD-sEMG Paper^66^	sEMG-RD Paper^64^
Channels	8	10	8	8	16	21	7	32	16
ADC	8 bits	10 bits	8/12 bits	24 bits	16 bits	24 bits	14 bits	16 bits	12 bits
Sampling rate	200 sps	1,000 sps	1,000 sps	500 sps	2,000 sps	1,067 sps	1,000 sps	1,000 sps	2,000 sps
Bandwidth	5–100 Hz	20–500 Hz	20–500 Hz	0–250 Hz	2–1,000 Hz	30–350 Hz	30–400 Hz	10–450 Hz	20–850 Hz
Electrode material	stainless steel	electroless nickel immersion gold	silver-coated stainless steel	stainless steel	gold-plated copper	beryllium copper	Ag/AgCl	gold-plated PCB pad	gold-plated brass
Contact dimensions	100 mm^2^	50 mm^2^	66 mm^2^	–	48 mm^2^	Φ2.3 mm ball	<243 mm^2^	50.27 mm^2^	33 mm^2^
Input reference noise	–	2.2 μV	–	–	2.4 μV	2.25 μV	–	2.4 μV	2.46 μV
Gain	–	–	1,200	12	192	–	250–4,000	192	190
IMU	9-axis	9-axis	9-axis	6-axis	9-axis	–	–	–	9-axis
Transmitter	BLE4.0	enhanced shockburst	BLE4.1	WIFI	BLE5.1	wired	BLE4.2	2.4 GHz	BLE5.0
Weight	93 g	62 g	80 g	100 g	70 g	100 g	–	–	–
Battery capacity	520 mAh	100 mAh	200 mAh	980 mAh	200 mAh	wired	175 mAh	650 mAh	–
Power consumption	16 h	6 h	100 mW	4–6 h	6 h	50 mW	60 h	49.5 mW	4 h
Price	200 USD	150 USD	1,250 USD	729 USD	200 USD	–	–	–	–

The hardware design of sEMG armbands is crucial to overall performance.^87^ Among various parameters, the number of acquisition channels strongly influences spatial resolution and decoding robustness. The human forearm contains six muscle groups: flexor carpi ulnaris, flexor digitorum, flexor carpi radialis, extensor carpi radialis, extensor digitorum, and extensor carpi ulnaris. Each gesture mainly involves a subset of these muscles, and strong correlations exist between gestures and the sEMG signals from different forearm muscles.^88^^,^^89^ Increasing the number of channels provides denser spatial sampling, improving the likelihood of capturing dominant muscles and enhancing recognition accuracy.

Electrode material and geometry strongly affect sEMG signal fidelity^90^^,^^91^^,^^92^ by influencing electrode-skin impedance and charge transfer. High-conductivity and electrochemically stable materials, such as Ag/AgCl or Au,^79^ reduce impedance and baseline drift, improving the signal-to-noise ratio (SNR), while gold-coated copper electrodes offer a cost-effective alternative. Electrode area involves a trade-off between impedance and spatial resolution: larger areas reduce noise, whereas smaller electrodes provide higher resolution but are more sensitive to motion artifacts. An earlier study^93^ showed that the electrode area and alignment relative to muscle fibers significantly impact signal quality. Inter-electrode spacing further determines crosstalk levels; experiments spanning 5–40 mm^94^ identified 10 mm as optimal for minimizing crosstalk. In addition, flexible electrodes based on carbon and polymer materials^95^^,^^96^ enhance mechanical conformity, biocompatibility, and long-term wearability for sEMG armbands.

sEMG signals are analog and must be digitized by an ADC before processing by a microcontroller. The ADC resolution determines the smallest detectable voltage change. Higher resolution converters reduce quantization noise and preserve finer waveform details,^97^ enhancing the accuracy of subtle gesture recognition.

Sampling rate directly affects the temporal resolution and fidelity of sEMG signals, influencing gesture recognition accuracy. Higher rates preserve rapid muscle activation dynamics and fine-grained spectral features essential for robust classification, whereas low rates cause aliasing, information loss, and degraded feature representation. sEMG frequency content mainly lies between 20 and 500 Hz.^98^^,^^99^^,^^100^ Angkoon Phinyomark et al.^101^ showed that reducing the sampling rate from 1,000 to 200 Hz sharply decreased discriminative information for sEMG-based control. High-frequency components significantly improve recognition of complex hand and finger movements, particularly in transradial amputees, with gains exceeding 10%. This is because a 1000 Hz sampling rate meets the Nyquist theorem’s minimum requirement for faithfully reconstructing 500 Hz sEMG signals.^102^

Filtering is essential for maintaining sEMG signal purity, as noisy signals hinder accurate gesture discrimination.^103^ Raw sEMG is weak and contaminated by motion artifacts, 50/60 Hz power line interference, electrocardiographic signals, and system noise.^104^^,^^105^ After electrode detection, signals are processed through preamplification, high- and low-pass filtering, and sometimes software-based algorithms to retain relevant muscle activity while suppressing noise.^106^^,^^107^ Future developments in adaptive filtering, combined with adjustable sampling rates and gain, are expected to further enhance sEMG fidelity, improving gesture recognition accuracy and robustness across tasks and users.

Incorporating IMUs provides complementary motion and posture information that helps disambiguate gestures with similar sEMG patterns.^108^^,^^109^^,^^110^ Meanwhile, Bluetooth Low Energy (BLE) is widely adopted in sEMG armbands due to its low power consumption and stable short-range transmission.^111^^,^^112^ Overall, ongoing hardware advances are driving sEMG armbands toward low-power, low-cost, and ergonomic designs, alongside growing interest in multimodal integration for improved robustness and recognition performance.

## Current research and future trends in multimodal fusion sensing for sEMG armbands

Although multimodal fusion has become a research hotspot,^113^ most armband systems in practical applications still primarily combine sEMG with IMU. This limits the information dimension and makes it difficult to fully perceive user intent or adapt to complex interaction scenarios.^114^ This chapter reviews recent progress in multimodal fusion for gesture recognition. It focuses on integration methods and collaborative perception mechanisms of sEMG with IMU, force myography (FMG), magnetomyography (MMG), sonomyography (SMG), near-infrared spectroscopy (NIRS), light myography (LMG), electrical impedance tomography (EIT), and other signals, analyzes representative achievements and advantages of various fusion schemes, and discusses development bottlenecks and future trends in sEMG armband multimodal fusion.

### Multimodal fusion of sEMG and IMU

IMU typically includes an accelerometer, gyroscope, and magnetometer, capable of capturing the arm’s motion state, angular velocity, acceleration, and orientation in real time.^115^ Integrating an IMU into an sEMG armband can compensate for sEMG’s spatial limitations, enhancing the system’s ability to recognize dynamic gestures.^116^ Wenjin Tao et al.^117^ combined sEMG signals from the Myo armband with IMU data, extracting features via discrete Fourier transform and CNNs to efficiently recognize six assembly tasks performed by workers. In two validation experiments, the model reached recognition accuracies of 98% and 87%, demonstrating that sEMG-IMU fusion significantly enhances activity recognition under complex conditions. Jiang et al.^118^ designed a wristband for real-time gesture recognition using sEMG-IMU fusion, leveraging IMU’s complementary perception of motion to efficiently recognize multiple airborne and contact gestures. Experimental results showed that the recognition accuracy stably ranged from approximately 89% to 93%, confirming IMU’s key role in improving wrist gesture recognition. Additional studies^119^^,^^120^^,^^121^^,^^122^ similarly show that fusion strategies enhance recognition accuracy and robustness. IMU chips are typically low power, compact, easy to integrate, and low cost, facilitating their widespread adoption in sEMG armbands.

### Multimodal fusion of sEMG and FMG

FMG is an alternative sensing technology to sEMG that recognizes movements by detecting fluctuations in skin surface pressure caused by local muscle bulging and volume changes from muscle activity.^123^ Compared with sEMG, which is sensitive to weak bioelectrical signals, FMG is more robust against electromagnetic interference, sweat, and changes in skin resistance and offers advantages such as low cost and simple structure.^124^^,^^125^

Jiang et al.^126^ proposed an integrated EMG-FMG fusion armband incorporating eight co-located sEMG-FMG sensing units capable of simultaneously acquiring electrophysiological and mechanical signals from the same muscle locations. Each unit is compact (11 × 13 × 6 mm), weighs only 0.9 g, and is evenly distributed along the forearm. The sEMG component uses silver foil electrodes and a high-precision ADS1299 acquisition chip, providing 24-bit resolution at a 1,000 Hz sampling rate, and employs common ground referencing and right leg drive technology to enhance anti-interference capability. The FMG component uses TakkTile pneumatic sensors embedded in rubber, sampled at 60 Hz, with good linear response and low hysteresis. Experimental results show that for recognizing American Sign Language digits 0–9, the fusion system achieved 91.6% accuracy, significantly higher than sEMG (81.5%) or FMG (80.6%) alone. This demonstrates the complementary nature of EMG and FMG in perception.

Zhang et al.^127^ proposed a co-located multimodal myoelectric armband integrating sEMG with pneumatic force myography (pFMG). The system uses flexible 3D-printed air chambers as the pFMG module, with three sEMG electrodes placed on top of each chamber to acquire electrophysiological and mechanical signals from the same muscle region without interference. The armband includes eight pairs of EMG-pFMG sensors, with signals synchronously acquired at 1,000 Hz and transmitted via USB, ensuring accurate sampling and a fast system response. In a hand gesture recognition experiment involving 7 gestures and 14 participants, the fusion system achieved 94.6% accuracy, higher than sEMG (87.95%) or pFMG (80.11%) alone, with an improvement of over 15%.

Previous studies^128^^,^^129^^,^^130^^,^^131^^,^^132^^,^^133^^,^^134^ have also indicated that sEMG-FMG fusion improves gesture recognition performance and robustness compared to single-modality approaches. From a sensing perspective, FMG provides a mechanically based, low-cost, and hardware-efficient measurement of muscle activity, but its signal quality depends strongly on sensor tightness and placement, leading to reduced repeatability.^135^^,^^136^^,^^137^^,^^138^ Consequently, FMG is better positioned as a complementary modality to sEMG rather than a standalone solution.

### Multimodal fusion of sEMG and MMG

Skeletal muscle activity generates measurable magnetic fields, leading to the concept of MMG.^139^^,^^140^ An earlier study^141^ tested a wearable MMG sensor system for recognizing 9 static gestures across 30 intact users. While the system achieved 95.4% average accuracy, each magnetic sensor costs over 1,000 USD, and all experiments were conducted inside a magnetically shielded room. Some other studies^142^^,^^143^^,^^144^^,^^145^^,^^146^^,^^147^ have also explored MMG for muscle activity detection, but they faced similar issues such as strong environmental interference and high cost, which greatly limit wearable deployment. Given these unresolved challenges, current MMG research remains primarily a laboratory-stage conceptual exploration.

### Multimodal fusion of sEMG and SMG

Since ultrasound was first used for prosthetic control in 2006, ultrasound-based gesture recognition has developed rapidly.^148^ It uses high-frequency sound waves to image internal structures, accurately mapping forearm anatomy and detecting muscle morphological changes. Recognizing gestures by monitoring ultrasound images is thus feasible.^149^ sEMG alone often struggles with fine finger movements,^107^ whereas ultrasound can better identify precise finger motions.^150^^,^^151^^,^^152^ Consequently, some studies have integrated ultrasound sensors into sEMG armbands to provide more comprehensive muscle activity information.

Xia et al.^153^ proposed a multi-channel portable hybrid sEMG/A-mode ultrasound (AUS) human-machine interface system. This compact armband integrates two sensing modalities to simultaneously acquire electrophysiological and morphological information from the same muscle region. The linear array structure places the ultrasound transducer between two sEMG electrodes, ensuring both sensors act on the same muscle group. An aluminum foil shielding layer suppresses electromagnetic interference from ultrasound excitation. The ultrasound module is built with TI’s LM96570 beamformer and ST’s STHV748 high-voltage waveform amplifier. The system is controlled by a dsPIC33EP512MU814 DSP from Microchip. Experimental results demonstrate significant advantages in gesture recognition. Compared with using ultrasound alone, the fusion of sEMG and AUS features improved recognition accuracy by 4.85%; compared with sEMG alone, the improvement reached 20.6%. However, the SMG unit costs over 200 USD and consumes more than 850 mW.

Zou et al.^154^ developed a wearable multimodal data acquisition platform integrating an sEMG armband (OYMotion Technologies, China), an A-mode ultrasound transducer (Shantou Ultrasonic Electronics Company), and a custom hand force sensor. These components achieve synchronized acquisition via the NI USB-6008 module. Experimental results showed that, compared with using only sEMG or ultrasound, the fusion model reduced the normalized mean square error (NMSE) of hand force estimation by 97.7% and 38.92%, respectively, performing well in both healthy participants and some stroke patients. Some papers^155^^,^^156^^,^^157^ also reported that fusing sEMG and ultrasound improves gesture recognition accuracy and robustness.

Ultrasound can sense deep muscle activity and complement the electrophysiological information provided by sEMG.^158^^,^^159^ However, these benefits come at the cost of high power consumption, bulky hardware, and elevated system complexity and cost, making the integration of SMG into sEMG armband-based wearables highly challenging.^160^^,^^161^

### Multimodal fusion of sEMG with other sensing modalities

In addition to commonly used modalities, researchers have explored integrating sEMG with other sensing types. Guo et al.^162^ developed a multi-channel wireless armband combining sEMG and NIRS. The sEMG module uses an INA326 instrumentation amplifier and an AD8603 operational amplifier for two-stage amplification with band-pass filtering. The NIRS module integrates tri-wavelength LEDs (Light Emitting Diodes), with a photodiode (OPT101) and transimpedance amplifier converting scattered light into voltage output. LEDs are powered via pulsed signals using a DM11A driver to prevent skin damage. Experimental results show that fusing sEMG and NIRS features improves the classification accuracy of 13 gestures compared to using sEMG or NIRS alone. However, the armband suffers from severe energy constraints: the optical light source alone consumes up to 680 mW, and the estimated system cost is approximately USD 130. Moreover, similar limitations have been observed across other NIRS-based studies^163^^,^^164^^,^^165^^,^^166^ on muscle activity analysis, including high power consumption and strong sensitivity to ambient light, which significantly restrict the practicality of integrating NIRS into long-term wearable armband systems.

Guan et al.^167^ proposed a high-density light myography (HDLMG) armband system for real-time muscle signal decoding in prosthetic control. LMG detects local blood flow and tissue changes by penetrating the skin with multi-wavelength light and leveraging tissue absorption and scattering characteristics,^168^ enabling motion recognition. Using this system, offline experiments involving 10 gesture types showed that models based on random forests and CNNs achieved accuracies of 94.11% and 95.87%, respectively. However, in real-time experiments, recognition accuracy decreased over time, dropping to 61.77%–34.66% across the 10 classes. Furthermore, the system entails a high system cost of approximately USD 400 and a total power consumption exceeding 1 W. The optical nature of LMG makes it sensitive to sensor placement, ambient light, and perspiration, resulting in poor long-term stability.^18^ To address these challenges, researchers have explored strategies including background illumination compensation, sweat-based signal correction,^169^ and spatially adaptive algorithms; these approaches remain largely experimental. LMG is still immature, and despite its compact optical sensors, substantial technical limitations currently hinder its practical integration into sEMG armbands.

Zhang et al.^170^ proposed Tomo, an EIT-based gesture recognition armband that reconstructs subcutaneous impedance images from eight electrodes, achieving low power consumption (∼50 mW) and high recognition accuracy (93%). However, the system is highly sensitive to electrode placement and exhibits poor repeatability. Although adaptive methods^171^ have been proposed to mitigate drift caused by re-wear, their effectiveness is limited, severely restricting practical use in wearable armband systems.

In summary, sEMG armbands remain the core modality in wearable gesture-recognition systems due to their high temporal resolution, non-invasive acquisition, and rich signal content. Multimodal fusion has become a clear trend, but it inevitably increases system size, power consumption, crosstalk, and algorithmic complexity.

## Discussion

sEMG signals, which directly capture muscle electrical activity, remain one of the most informative biosignals for gesture recognition. Recent progress in hardware—such as high-density electrode arrays, low-noise multi-channel acquisition circuits, and improved analog front-ends—together with advances in deep learning has substantially enhanced decoding accuracy and robustness. A key driver of these improvements is the increasing scale and diversity of training datasets; recent large-cohort studies (e.g., those by Meta) demonstrate that when broad, user-spanning datasets are combined with adaptive neural decoders, sEMG systems can achieve both high accuracy and strong cross-user performance, addressing limitations seen in earlier small-scale studies. Meanwhile, multimodal fusion—integrating sEMG with inertial, force, optical, or ultrasound sensing—has shown clear benefits under dynamic or noisy conditions, yet each modality presents distinct trade-offs in cost, power, and integration feasibility. A systematic understanding of these trade-offs is therefore essential for guiding the design of next-generation sEMG armband systems.

Tables 3 and 4 provide a systematic comparison of multiple sensing modalities. The IMU module is characterized by compact size, low power consumption, and low cost. For example, the 3DC and α armbands utilize the ICM90248 chip,^50^^,^^58^ which measures only 3 × 3 mm, consumes 2.65 mW, and costs less than 10 USD per unit. The built-in Digital Motion Processor (DMP) can directly output attitude estimation results, thereby reducing the computational burden on the MCU. With a sampling rate of 50 Hz, IMUs can effectively capture dynamic gestures and enable recognition within 100 ms,^117^^,^^118^ making them an indispensable sensing component in sEMG-based systems.

**Table 3:** Comparison of advantages and disadvantages of multimodal systems

Modalities	Full name	Principles	Advantage	Disadvantage
sEMG	surface electromyography	measures electrical potentials generated by muscle fibers during contraction	intuitive; rich information; simple; low cost; mature technology	weak; easily interfered; only capable of measuring superficial muscles
IMU	inertial measurement unit	measures acceleration, angular velocity, and infers attitude and motion	compact; low power; low cost; motion measurement	does not provide measurements of muscle activation or static postures
FMG	force myography	measures changes in muscle shape and force through pressure or deformation on the skin surface	simple; EMI resistant; low cost	sensitive to tightness; only measures surface signals
MMG	magnetomyography	measures magnetic field changes generated by muscle electrical activity	non-contact; physiologically stable; high resolution; deep-muscle measurement	sensitive to environment; sensor cross-interference; high cost; immature technology
SMG	sonomyography	measures tissue motion using high-frequency ultrasound reflections for real-time imaging	crosstalk resistant; deep-muscle measurement; high resolution	high power consumption; large size; high cost; heavy weight
NIRS	near-infrared spectroscopy	measures changes in reflected near-infrared light caused by variations in blood volume and oxygenation	high penetration; high resolution	high power consumption; sensitive to tissue thickness; sensitive to ambient light
LMG	light myography	measures tissue changes and blood flow via multi-wavelength light absorption and scattering	high penetration; simple; compact	high power consumption; sensitive to environment; sensitive to physiological state
EIT	electrical impedance tomography	measures subcutaneous impedance to reconstruct internal tissue images	deep-muscle measurement; high resolution	sensitive to electrode placement and skin contact; sensitive to electromagnetic

**Table 4:** Practical trade-offs of sensing modalities for wearable armbands

Modalities	Accuracy	Cost	Power	Robustness	Integration difficulty
sEMG	high	low	low	medium	low
IMU	medium	low	low	high	low
FMG	high	low	medium	medium	low
MMG	high	high	medium	high	high
SMG	high	high	high	medium	high
NIRS	medium	low	high	low	high
LMG	medium	high	high	low	high
EIT	medium	low	low	low	medium

FMG, benefiting from its resistance to electromagnetic interference, compensates for one of the main drawbacks of sEMG. The EMG-FMG sensing unit developed by Jiang et al.^126^ measured 11 × 13 × 6 mm and weighed 0.9 g, employing a TakkTile pressure sensor costing about 30 USD. Experimental results showed that fusing FMG and sEMG at 60 Hz improved gesture recognition accuracy by approximately 10% compared with sEMG alone, using only 200 ms signal segments. Although FMG is sensitive to strap tightness and requires calibration before use, it remains a valuable complement to sEMG for enhancing signal robustness.

Accurate recognition of fine motor control requires information from both superficial and deep muscle layers, which sEMG alone cannot fully capture. Ultrasound-based sensing (SMG) enables deep muscle detection, providing richer physiological cues for gesture decoding. An earlier study^157^ showed that fusing sEMG with SMG improved accuracy by 20.6%, underscoring the value of deep-muscle sensing. However, the SMG unit costs over 200 USD and consumes more than 850 mW. Its hardware consisted of three separate PCBs (Printed Circuit Boards) connected to the armband via four external cables, making integration into an sEMG armband impractical. Although SMG hardware optimization is ongoing, the gap between current designs and the requirements for compact, low-power, fully wearable systems remains substantial.

EIT and MMG have also been investigated for their ability to capture information related to deeper muscle activities. The eight-channel EIT armband proposed by Zhang et al.^170^ achieved 90% average accuracy for 11 static gestures with only 50 mW power consumption and a cost of about 40 USD. However, its performance shows poor repeatability and generalization. The device is also relatively bulky and relies on multiple external cables, and it functions solely as an EIT system without integrating sEMG, which further limits its practicality for wearable armband applications. In addition, an earlier study^141^ reported an MMG armband achieving 95.4% average accuracy for 9 static gestures across 30 intact users; each magnetic sensor costs more than 1,000 USD, and all experiments were conducted inside a magnetically shielded room, conditions far removed from practical deployment.

Optical modalities such as NIRS and LMG remain largely impractical for wearable use due to their substantial power consumption. For example, one sEMG-NIRS armband reported in a previous study^162^ cost approximately 130 USD, yet its light source alone consumed about 680 mW, and three 3.7 V/600 mAh batteries could sustain only around 2.5 h of operation. LMG suffers from similar limitations. A representative LMG system proposed in another study^167^ cost about 400 USD and incorporated 40 phototransistors, driving the total power consumption to over 1 W—well beyond what compact, battery-powered wearables can tolerate.

In summary, IMU is an essential and mature modality for sEMG-based systems, while FMG serves as a valuable supplement. SMG is mainly limited by its high power consumption and cost. In contrast, EIT, MMG, NIRS, and LMG remain at the proof-of-concept stage and are not yet suitable for integration into sEMG-based armband systems.

Beyond multimodal fusion, further exploration of device-level innovations—such as flexible materials, high-density electrode architectures, and ergonomic design—can also improve system performance, usability, and long-term reliability.

(1) Flexible, skin-conformal materials represent one of the key directions for future development of sEMG armbands, enhancing electrode-skin contact stability, reducing motion artifacts, and supporting long-term wearability. Advanced materials—including liquid-metal interconnects, graphene, carbon nanotubes, and conductive polymers—enable durable, biocompatible, and mechanically resilient designs.^172^ Incorporating such materials can improve the performance of all integrated sensors, strengthen the advantages of multimodal fusion, and enhance signal fidelity and robustness under dynamic and real-world conditions.
(2) High-density electrode arrays are also a key direction for the advancement of sEMG armbands, offering enhanced spatial resolution of muscle activation patterns. The increased density of electrodes captures richer and more detailed signal information, which enhances the integrative capacity of multimodal systems. Such architectures contribute to more robust and reliable neuromuscular monitoring across diverse users and dynamic conditions, reinforcing the performance and applicability of multimodal fusion in wearable myoelectric devices.
(3) Ergonomic design constitutes a crucial direction for sEMG armband development, as user anatomical variability can affect sensor placement, signal stability, and system reliability. Armbands with optimized geometry and layout ensure consistent electrode contact and comfort across diverse forearm shapes and muscle distributions. By supporting stable signal acquisition for a wide range of users, such designs enhance the adaptability and generalizability of multimodal fusion, enabling robust performance in real-world scenarios and facilitating broader practical application.

## Conclusion

This review systematically summarizes the current research status of sEMG armbands in the field of gesture recognition. By analyzing existing sEMG armband products and evaluating the performance of various modalities, two main developmental trends in technology can be identified. First, hardware architectures are becoming increasingly integrated and intelligent, accompanied by continuous optimization of filtering and signal processing algorithms to enhance data quality. While ensuring reliable gesture recognition functionality, it is essential to balance power consumption, cost-effectiveness, and wearing comfort, striving for an optimal integration of performance and ergonomic considerations. Second, future armbands for gesture recognition will be built around sEMG as the core modality, integrated with readily implementable sensing technologies such as IMU and FMG to establish foundational fusion. Meanwhile, SMG and other deep-sensing modalities may be introduced gradually, provided that their power, size, and cost constraints are effectively addressed. The ultimate goal is to construct high-performance, power-efficient, and cost-effective multimodal systems capable of meeting the increasingly complex demands of gesture recognition and human-machine interaction.

## Limitations

Despite recent advances, sEMG armband systems still face key challenges that hinder their robustness and real-world applicability, particularly in terms of cross-subject generalization, long-term stability, and electrode placement.

Cross-subject generalization remains a major challenge in sEMG-based gesture recognition. Due to inter-subject variability in muscle morphology, skin properties, and activation patterns, the performance of pre-trained models often deteriorates when applied to new users. Current approaches to mitigate this issue typically involve fine-tuning models with extensive calibration data from new users, which is cumbersome and time-consuming. A recent study by Ye et al.^173^ proposed a dynamic domain generalization (DDG) method that enables accurate gesture recognition for new subjects without any calibration. This method leverages a meta-adjuster to dynamically generate template coefficients for network parameters, adapting temporal, spatial, and spatiotemporal features, as well as normalization layers, to individual differences. In addition, Zhang et al.^174^ introduced a multi-source synchronized domain adaptation framework that aligns the feature spaces of multiple training users with a new user, improving cross-subject knowledge transfer while preserving combined source data. Collectively, these studies suggest that future sEMG armband systems should adopt adaptive architectures capable of accommodating inter-subject variability with minimal or no user-specific calibration.

Long-term stability is also a critical challenge for sEMG armband systems. Over time, changes in electrophysiological factors—particularly muscle fatigue, along with perspiration and electrode displacement—can lead to a significant decline in gesture recognition accuracy. Phinyomark et al.^175^ report that, in long-term testing, the classification performance of forearm-based pattern recognition systems can decrease by up to 55%, with muscle fatigue identified as a major contributor. To address this issue, adaptive algorithms that continuously monitor signal variations and dynamically update classification model parameters are necessary to maintain system reliability.^176^ In addition, Pan et al.^177^ proposed a domain adaptation-based approach that aligns EMG feature distributions across non-fatigue and fatigue states, effectively mitigating the impact of muscle fatigue on signal decoding and enhancing system robustness. Overall, ensuring long-term stability is essential for practical sEMG armband applications, particularly in clinical settings, where consistent and reliable sEMG performance is critical for patient safety, rehabilitation outcomes, and real-world usability.

In addition, electrode placement plays a crucial role in determining the overall performance and robustness of sEMG-based gesture recognition systems. Islam et al.^178^ demonstrated that recognition performance varies significantly with electrode positions along the forearm muscles in both circumferential and longitudinal directions. Multiple electrode arrays placed near the elbow joint achieved substantially higher SNRs and signal-to-motion artifact ratios compared to other regions. Furthermore, Botros et al.^179^ conducted a comprehensive comparison between wrist and forearm EMG signals and ultimately recommended the wrist as a more practical placement due to its superior signal quality and user comfort. However, for transradial amputees with limited residual forearm length, wrist-based placement may not be feasible. Therefore, future sEMG armband designs should carefully consider sensor positioning and spatial configuration, as electrode placement directly influences signal quality, motion robustness, user comfort, and system adaptability across different anatomical and clinical conditions.

Current sEMG armband systems have achieved high accuracy under controlled conditions; however, the primary challenge remains robustness across users, sessions, and device configurations. Enhancing cross-subject generalization and long-term stability is therefore critical for practical deployment. Advances in flexible materials, high-density electrode arrays, ergonomic design, multimodal fusion, adaptive filtering, and model optimization can further improve signal fidelity and system adaptability. Moreover, the use of standardized, publicly accessible benchmark datasets, such as sEMG-RD and NinaPro, or the establishment of new large-scale public datasets is essential for reliably evaluating robustness and ensuring reproducible comparisons. Collectively, these strategies are expected to guide next-generation multimodal armbands toward stronger robustness and broader real-world applicability.

## Acknowledgments

The work described in this paper was supported by the National Natural Science Foundation of China Youth Science Fund (51705475).

## Author contributions

Conceptualization, R.Z. and Y.H.; methodology, R.Z., Y.H., and X.Y.; investigation, R.Z., H.D., X.Y., and L.D.; writing – original draft, R.Z. and L.D.; writing – review and editing, R.Z. and Y.H.; funding acquisition, Y.H. and H.Z.; resources, Y.H. and H.Z.; supervision, Y.H., X.Y., and H.D.

## Declaration of interests

The authors declare no competing interests.

## Declaration of generative AI and AI-assisted technologies in the writing process

This manuscript was written entirely by the authors without the use of generative AI or AI-assisted technologies.
